# Dark horse target Claudin18.2 opens new battlefield for pancreatic cancer

**DOI:** 10.3389/fonc.2024.1371421

**Published:** 2024-03-06

**Authors:** Qian Xu, Caiyan Jia, Yan Ou, Chuanxiu Zeng, Yingjie Jia

**Affiliations:** ^1^ Department of Oncology, First Teaching Hospital of Tianjin University of Traditional Chinese Medicine, Tianjin, China; ^2^ Department of Oncology, National Clinical Research Center for Chinese Medicine Acupuncture and Moxibustion, Tianjin, China

**Keywords:** pancreatic cancer, Claudin18.2, targeted therapy, immunotherapy, trials

## Abstract

Pancreatic cancer is one of the deadliest malignant tumors, which is a serious threat to human health and life, and it is expected that pancreatic cancer may be the second leading cause of cancer death in developed countries by 2030. Claudin18.2 is a tight junction protein expressed in normal gastric mucosal tissues, which is involved in the formation of tight junctions between cells and affects the permeability of paracellular cells. Claudin18.2 is highly expressed in pancreatic cancer and is associated with the initiation, progression, metastasis and prognosis of cancer, so it is considered a potential therapeutic target. Up to now, a number of clinical trials for Claudin18.2 are underway, including solid tumors such as pancreatic cancers and gastric cancers, and the results of these trials have not yet been officially announced. This manuscript briefly describes the Claudia protein, the dual roles of Cluadin18 in cancers, and summarizes the ongoing clinical trials targeting Claudin18.2 with a view to integrating the research progress of Claudin18.2 targeted therapy. In addition, this manuscript introduces the clinical research progress of Claudin18.2 positive pancreatic cancer, including monoclonal antibodies, bispecific antibodies, antibody-drug conjugates, CAR-T cell therapy, and hope to provide feasible ideas for the clinical treatment of Claudin18.2 positive pancreatic cancer.

## 1 Introduction

**Figure 1:**
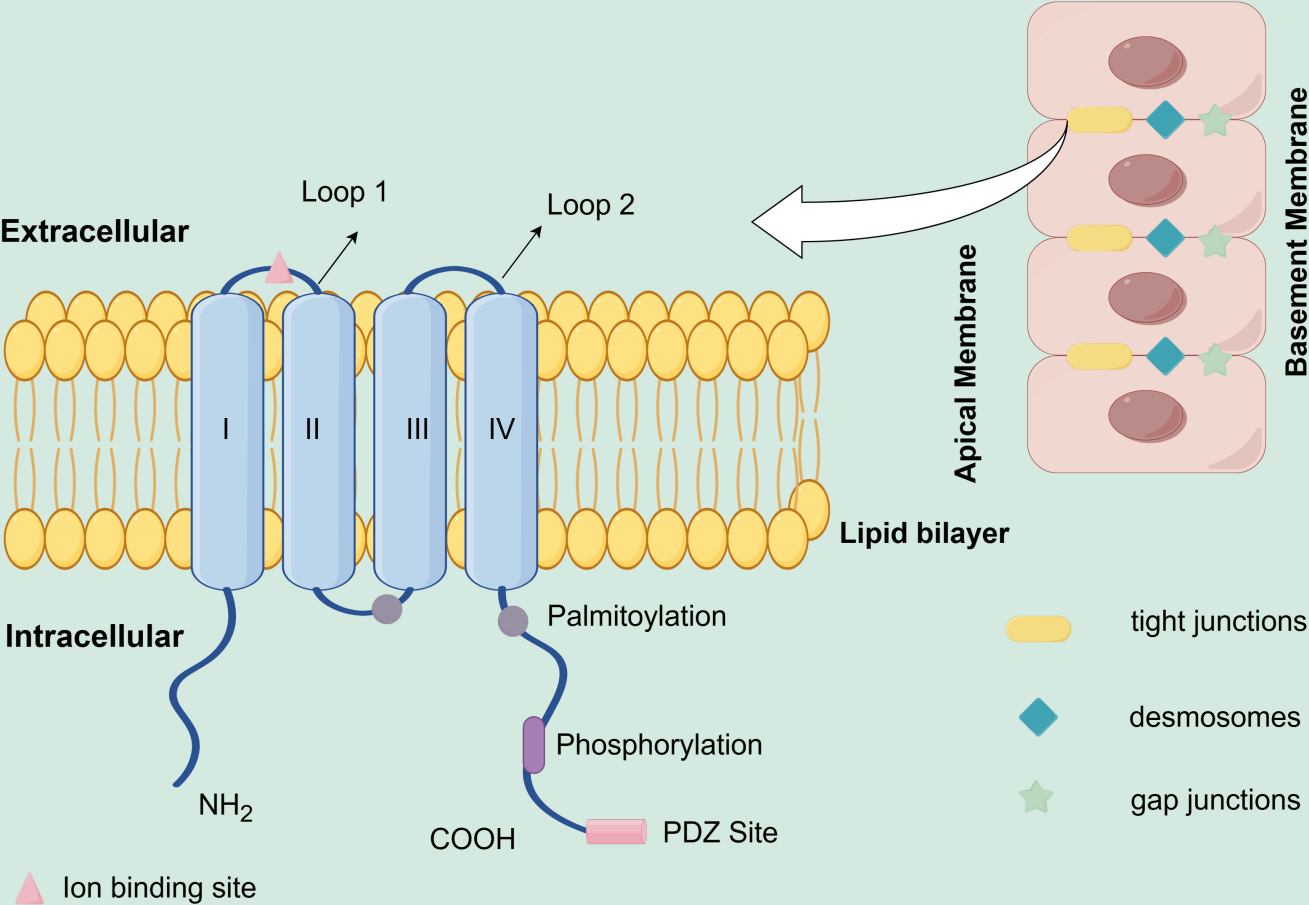
Schematic diagram of Claudins protein structure. (By Figdraw).

Claudins are important proteins in normal tissue tight junctions (TJ), which are related to the formation of epithelial and epidermal tight junction proteins (1), and were first discovered and named by researchers Mikio Furuse and Shoichiro Tsukita of Kyoto University in Japan in 1998. The name “Claudin” originated from the Latin word “claudere (to close)” (2). Three main functions of Claudins are intracellular signaling, fence, and barrier. While the function of fence divides the apical and basolateral domains and controls substance mobility in the plasma membrane, the function of barrier is the capacity to control the paracellular permeation of ions, water, immune cells, and macromolecules, selectively (3–5). Claudins include at least 27 family members, 4 transmembrane structural domains, and 2 extracellular loops (Schematic diagram of Claudins protein structure, **Figure 1**), which allow the Claudins proteins to effectively sustain the polarity of epithelial and endothelial cells, thereby effectively regulating paracellular permeability and conductance (6). Claudin18 is one of the Claudins (CLDN) proteins family, which has two different isoforms, Claudin18.1 and Claudin18.2, due to the optional exons 1a and 1b (7). Claudin18.2 is the highly targeted marker protein with tissue-specific expression. The expression of Claudin18.2 in normal tissues is restricted to differentiated gastric mucosal epithelial cells, which results that it is masked and is mostly untouched by intravenous antibodies. However, if the tissues are cancerous, changes in cell polarity expose the epitope of Claudin18.2 to the cell surface and increase the expression (8). Claudin18.2 is expressed in many tumors, including gastric cancer, pancreatic cancer, esophageal cancer, ovarian cancer, etc. (8–11). In a study of 414 cancer patients with immunohistochemistry (IHC) analysis to assess Claudin18.2 expression in different tumor types, a total of 4.1% (N=17) of subjects were Claudin18.2 positive, including pancreatic cancer (16.7%, 1/6), gastric cancer (14.1%, 12/85), colorectal cancer (0.9%, 2/203), biliary tract cancer (6.3%, 1/16), and genitourinary/miscellaneous (2.2%, 1/46) (12). The expression of Claudin18.2 is not only in the primary tumor, but also in metastases. In one study, pancreatic ductal adenocarcinoma (PDAC), which is the most common kind of pancreatic cancers, showed 59.2% (103/174) Claudin18.2 expression, of which 54.6% were strongly expressed (N=95). In the lymphatic metastasis, 69.4% (N=34) expressed Claudin18.2 with strong staining. Whereas the number of tumor cells in the samples expressing Claudin 18.2 was positively linked with the staining intensity (RS = 0.871, p<0.001). The results are similar in liver metastasis samples. 65.7% of samples (N=23) expressed Claudin18.2 and all positive samples showed 2+ or higher staining intensity (13). Jiang H et al. finds that the expression of Claudin18.2 in the metastatic lesions is in line with the expression in the primary cancer tissues (14). And the expression of Claudin18.2 was more obvious in the disease progression. Samples from lymph nodes and liver metastases showed expression that was either identical to or higher than that in the original lesion, and the high expression of Claudin18.2 were related to the poor prognostic factors of positive lymph nodes (15). The longer median progression-free survival (mPFS) is related to the lower Claudin18.2 expression level in gastric cancer (p = 0.047) (16). Researchers also revealed that claudin18 expression correlates with poor survival in patients with CRC. And the prognosis of patients with positive claudin18 expression was significantly poorer than in negative cases (P= 0.0106) (17). While Jun et al. found that in patients with Claudin18 expression, OS was longer than those without Claudin18 expression (5-year survival rate, 90.5% vs. 64.8%) (18). Another study also found that patients with high expression levels of claudin18 have longer survival time than those with low claudin18 expression (19). Overexpression of Claudin18.2 may activate related signaling pathways, like SPAK-p38 MAPK signaling pathway, epidermal growth factor receptor (EGFR)/extracellular signal-regulated kinase (ERK) signaling et al., which leads to tumor cell proliferation (20–22). Another study on human pancreatic cancer cells showed Claudin18 is predominantly controlled at the transcriptional level by specific protein kinase C signaling pathways and modified by DNA methylation (23). At present, the exact mechanism that Claudin18.2 impacts on distant metastasis and tumor lymph node metastasis is unclear, and it is possible that its unusual expression changes the structure and function of tight junctions between cells. The studies have also found that different expressions of Claudin have been shown to be significant in the prognosis of cancer. For example, claudin-1 is associated with colon cancer prognosis (24), claudin-18 is related to gastric cancer (GC) prognosis (25), and claudin-10 is relevant to hepatocellular carcinoma prognosis (26). In summary, Claudin18.2 protein is involved in the migration, differentiation, and proliferation of tumor cells. And because of its specific expression it may be used as the target of precise attack on tumors, so the value of Claudin18.2 in the diagnosis, treatment and prognosis of tumors should be further explored.

## 2 The double-edged sword role of Claudin18 in cancers

There are many antigens with dual roles in tumor microenvironment, for example, TGF-β, desmosomes, HMGB1 (a nuclear DNA-binding protein) and cancer testis antigens etc. (27–30). Claudin18 also has different effects in tumors, acting as a tumor suppressant in gastric and lung cancers, but playing a promoting role in other gastrointestinal tumors, such as pancreatic cancer, colorectal cancer, and esophageal cancer (31). At present, only a few articles have elucidated the mechanism of action of Claudin18 in different tumors, which needs further summary.

### 2.1 Inhibition of tumors

Oshima et al. found that the Ki-67 index was inversely correlated with the level of Claudin18 in the front of gastric cancer invasion, and the loss of Claudin18 was also shown to be associated with the proliferation and invasion of GC cells in subsequent cell experiments (32). Helicobacter pylori infection in mice attenuated the expression of Claudin18 in the early stage of GC development, leading to rapid tumor development, which may be related to the involvement of Claudin18 in the regulation of cytokines, Wnt and Notch signaling pathways (33). Zhou et al. found that Claudin18 can inversely regulate the activity of Yes-associated protein (YAP), thereby promoting cell proliferation and tumorigenesis (34). Claudin18.1 can inhibit yes-associated protein/tafazzin (Yap/Taz) and insulin-like growth factor 1 receptor (IGF-1R) signaling pathway, resulting in AKT inhibition (35, 36). It proved that Claudins is an important signaling center involved in cell proliferation and tumorigenesis. In Claudin18-deficient mice, the frequency of lung adenocarcinoma also increased accordingly (37).

### 2.2 Promotion of tumors

The cancer-promoting effects of Claudin18 are mostly manifested in its ectopically activated cancers. For bile duct carcinoma cells, the overactivation of EGFR leads to the activation of the downstream Ras/Raf/MEK/ERK cascade, which induces the expression of Claudin18, and the expression of Claudin18 can further induce ERK1/2 phosphorylation, forming a positive feedback loop, and playing a role in promoting cancer (21). In the normal esophageal squamous epithelium, Claudin18 is not expressed, but the expression of Claudin18 in the columnar epithelium of Barrett’s esophagus is doubled, and Claudin18 can reduce the permeability of tightly junction H^+^ ions, prevent its attack and destroy the esophageal squamous epithelium, thereby promoting tumorigenesis (38). Claudin18.2 is mainly regulated by the hypomethylation gene sequence CpG island promoter and the transcription factor cAMP-response element blinding protein (CREB) in its genetic coding sequence (8), and its overexpression may be related to signaling pathways, like PKC pathway, ERK/MAPK pathway, and HER2/HER3 pathway, which promote the proliferation of tumor cells (31, 39, 40). In addition, the high expression of Claudin18.2 can regulate cell polarity, disrupt the tight junctions of tumor cells, change the adhesion and plasticity of tumor cells, increase their ability to metastasize and infiltration, and increase the degree of malignancy of tumors (41).

## 3 Current research of Claudin18.2

At present, there are no products targeting Claudin18.2 on the market, but research on various types of drugs targeting Claudin18.2 is in full swing, of which the indications are mostly focusing on Claudin18.2 positive gastric cancer and pancreatic cancer. Immunotherapy for solid tumor targeting Claudin18.2 includes monoclonal antibody, bispecific antibody, antibody-drug conjugate (ADC), and CAR-T cell. For example, the first targeted development drug, Zolbetuximab (IMAB362), is an IgG1 monoclonal antibody that specifically links structurally chimeric to Clauidn18.2 on the surface of tumor cells. It causes antibody-dependent cellular cytotoxicity (ADCC), complement-dependent cytotoxicity (CDC), apoptosis and inhibits cell proliferation. Its powerful capacity to eradicate cancer cells and regulate illness has been effectively confirmed by preclinical research (42). CMG901, monoclonal antibody + monomethyl auristatin E (MMAE) drug conjugates, can efficiently target tumor cells by anti-Claudin18.2 antibodies and trigger endocytosis, which allows the small molecule toxin MMAE to enter tumor cells to provide antitumor effects (14). Here, we list the latest clinical trials in Claudin18.2-targeted agents developed in ClinicalTrials.gov (**Table 1**) for further research.

**Table 1:** Latest clinical trials in Claudin18.2-targeted agents in ClinicalTrials.gov.

Agent	Type of Agents	NCT Number	Indications	Status	Phase	References
CMG901	ADC	NCT04805307	Advanced Solid Tumor, Gastric Cancer, Gastroesophageal Junction Adenocarcinoma, Pancreatic Cancer	Recruiting	I	(43)
SYSA1801	ADC	NCT05009966	Advanced Solid Tumor, Gastric Cancer, Gastroesophageal Junction Cancer, Pancreatic Cancer	Recruiting	I	
LM-302	ADC	NCT05161390	Advanced Solid Tumor	Recruiting	I/II	
LM-302	ADC	NCT05994001	Biliary Tract Cancer	Recruiting	I/II	
LM302	ADC	NCT05934331	Gastric Cancer, Pancreatic Cancer	Recruiting	II	
LM-302	ADC	NCT05001516	Advanced Solid Tumor	Active, not recruiting	I	
EO-3021	ADC	NCT05980416	Pancreas Neoplasm, Stomach Neoplasm, Gastrointestinal Neoplasms, Digestive System Neoplasm, Neoplasms by Site, Neoplasms	Recruiting	I	
SKB315 for injection	ADC	NCT05367635	Advanced Solid Tumors	Recruiting	I	
RC118-ADC	ADC	NCT05205850	Advanced Solid Tumor	Recruiting	I/II	
RC118	ADC	NCT06038396	Advanced Solid Tumor	Recruiting	I/II	
TORL-2-307-ADC	ADC	NCT05156866	Advanced Solid Tumor, Gastric Cancer, Pancreas Cancer, Gastroesophageal Junction Adenocarcinoma	Recruiting	I	
AMG-910	Bispecific antibodies	NCT04260191	Gastric and Gastroesophageal Junction Adenocarcinoma	Completed	I	(44)
Q-1802	Bispecific antibodies	NCT04856150	Advanced Solid Tumors	Recruiting	I	
SG1906	Bispecific antibodies	NCT05857332	Locally Advanced Unresectable or Metastatic Solid Tumors	Recruiting	I	
QLS31905	Bispecific antibodies	NCT06041035	Solid Tumor	Not yet recruiting	I/II	
AZD5863	Bispecific Antibodies	NCT06005493	Gastric Cancer, Gastro-esophageal Junction Cancer, Pancreatic Ductal Adenocarcinoma, Esophageal Adenocarcinoma	Recruiting	I/II	
QLS31905	Bispecific antibodies	NCT05278832	Advanced Solid Tumors	Recruiting	I	
PM1032 injection	Bispecific antibodies	NCT05839106	Advanced Tumor	Recruiting	I/II	(45)
ASP2138	Bispecific antibodies	NCT05365581	Gastric Adenocarcinoma, Gastroesophageal Junction (GEJ) Adenocarcinoma, Pancreatic Adenocarcinoma	Recruiting	I	
IBI389	Bispecific antibodies	NCT05164458	Advanced Solid Tumors	Recruiting	I	
CAR-CLD18 T Cells	CAR-T	NCT03159819	Advanced Gastric Adenocarcinoma, Pancreatic Adenocarcinoma	Unknown	Not Applicable	(46)
Engineered CAR-T Cells	CAR-T	NCT03198052	Lung Cancer, Cancer	Recruiting	I	
CAR-CLDN18.2 T Cells	CAR-T	NCT03874897	Advanced Solid Tumor	Recruiting	I	(47)
CT041	CAR-T	NCT04581473	Gastric Adenocarcinoma, Pancreatic Cancer, Gastroesophageal Junction Adenocarcinoma	Recruiting	I/II	(48)
CT041	CAR-T	NCT04404595	Gastric Cancer, Pancreatic Cancer	Recruiting	I/II	(49)
CT041	CAR-T	NCT05911217	Pancreatic Cancer	Recruiting	I	
LCAR-C18S	CAR-T	NCT04467853	Advanced solid Tumors	Terminated	I	
LY011	CAR-T	NCT04966143	Pancreatic Cancer	Recruiting	I	
LY011	CAR-T	NCT04977193	Advanced Gastric Adenocarcinoma	Recruiting	I	
TAC01-CLDN18.2	CAR-T	NCT05862324	Metastatic Solid Tumor	Recruiting	I/II	
IBI345	CAR-T	NCT05199519	Solid Tumors	Completed	I	
Claudin18.2 CAR-T cells	CAR-T	NCT05620732	Advanced Pancreatic Carcinoma, Advanced Gastric Carcinoma	Recruiting	Not Applicable	
Claudin 18.2 CAR-T	CAR-T	NCT05472857	Gastric Cancer, Pancreatic Cancer, Advanced Ovarian Carcinoma, Gastroesophageal Junction Adenocarcinoma	Recruiting	I	
RD07 Cell Injection	CAR-T	NCT05284968	Solid Tumor	Not yet recruiting	I	
LB1908	CAR-T	NCT05539430	Gastric Cancer, Gastroesophageal-junction Cancer, Esophageal Cancer, Pancreatic Cancer	Recruiting	I	
XKDCT086	CAR-T	NCT05952375	Gastric Cancer	Recruiting	Not Applicable	
HEC-016	CAR-T	NCT05277987	Advanced Gastric/Esophagogastric Junction Adenocarcinoma, Pancreatic Cancer	Recruiting	I	
AZD6422	CAR-T	NCT05981235	Gastrointestinal Tumors	Not yet recruiting	I	
KD-496	CAR-T	NCT05583201	Gastric Cancer, Pancreatic Cancer, Solid Tumor	Recruiting	I	
Dual-targeting CLDN18.2 and PD-L1 CAR-T cells	CAR-T	NCT06084286	Advanced Solid Tumor	Not yet recruiting	I	
CT048	CAR-T	NCT05393986	Gastric Adenocarcinoma, Pancreatic Cancer, Gastroesophageal Junction Adenocarcinoma, Advanced Solid Tumors	Recruiting	I	
IMC002 injection	CAR-T	NCT05946226	Advanced Digestive System Tumor	Recruiting	I	
IMC008	CAR-T	NCT05837299	Advanced Solid Tumor	Recruiting	I	
Zolbetuximab (IMAB362)	Monoclonal antibodies	NCT03504397	Locally Advanced Unresectable or Metastatic Gastric or Gastroesophageal Junction (GEJ) Adenocarcinoma	Active, not recruiting	III	
Zolbetuximab (IMAB362)	Monoclonal antibodies	NCT03505320	Metastatic or Locally Advanced Unresectable Gastric or Gastroesophageal Junction (GEJ) Adenocarcinoma	Recruiting	II	
Zolbetuximab (IMAB362)	Monoclonal antibodies	NCT03653507	Locally Advanced Unresectable or Metastatic Gastric or Gastroesophageal Junction (GEJ) Adenocarcinoma	Active, not recruiting	III	
Zolbetuximab (IMAB362)	Monoclonal antibodies	NCT03816163	Pancreatic Cancer, Metastatic Pancreatic Cancer, Metastatic Pancreatic Adenocarcinoma	Recruiting	II	(50)
Zolbetuximab (IMAB363)	Monoclonal antibodies	NCT01630083	Adenocarcinoma of the Gastroesophageal Junction, Adenocarcinoma of Esophagus, Gastric Adenocarcinoma	Completed	II	
Zolbetuximab	Monoclonal antibodies	NCT06048081	Locally Advanced Unresectable Gastroesophageal Junction (GEJ) Adenocarcinoma Cancer, Locally Advanced Unresectable Gastric Adenocarcinoma Cancer, Metastatic Gastric Adenocarcinoma Cancer, Metastatic Gastroesophageal Junction (GEJ) Adenocarcinoma	Available		
AB011	Monoclonal antibodies	NCT04400383	Solid Tumors, Gastric Cancer, Pancreatic Adenocarcinoma	Active, not recruiting	I	(51)
TST001	Monoclonal antibodies	NCT04495296	Advanced Cancer	Recruiting	I/II	
TST001	Monoclonal antibodies	NCT04396821	Advanced Cancer, Gastric Cancer, Gastroesophageal-junction Cancer, Pancreatic Cancer	Recruiting	I/II	
TST001	Monoclonal antibodies	NCT05190575	Biliary Tract Neoplasms	Completed	II	
TST001	Monoclonal antibodies	NCT06093425	Gastric Cancer, Gastroesophageal-junction Cancer, Advanced Cancer	Not yet recruiting	III	
ASKB589	Monoclonal antibodies	NCT04632108	Malignant Solid Tumor	Recruiting	I/II	
89Zr-NY005	Monoclonal antibodies	NCT04989010	Solid Tumor	Unknown	Not Applicable	
ZL-1211	Monoclonal antibodies	NCT05065710	Advanced Solid Tumors	Recruiting	I/II	
IMAB362	Monoclonal antibodies	NCT01671774	Gastric Adenocarcinoma, Adenocarcinoma of the Gastroesophageal Junction, Adenocarcinoma of Esophagus	Completed	I	
SPX-101	Monoclonal antibodies	NCT05231733	Solid Tumor	Not yet recruiting	I	
TORL-2-307-MAB	Monoclonal antibodies	NCT05159440	Advanced Solid Tumor, Gastric Cancer, Pancreas Cancer, Gastroesophageal Junction Adenocarcinoma	Recruiting	I	
M108	Monoclonal antibodies	NCT04894825	Advanced Unresectable Solid Tumors	Recruiting	I	
NBL-015	Monoclonal antibodies	NCT05153096	Advanced Solid Tumors	Not yet recruiting	I	
MIL93	Monoclonal antibodies	NCT04671875	Advanced Malignancies	Recruiting	I	

## 4 Application of Claudin18.2 in pancreatic cancer

Pancreatic cancer (PC) is one of the deadliest tumors, which seriously threatens human health. Given that pancreatic cancer has no special clinical manifestations and its unique anatomical location, it is considered to be a “silent killer”. In 2020, there were more than 490,000 new cases and more than 460,000 deaths worldwide. Pancreatic cancer kills almost as many people as it does, and the highest incidence rates are found in Europe and Northern America (26). With the lowest 5-year survival rate of any cancer type (10%), PC is also expected to become the second leading cause of cancer-related mortality in the United States by 2030 (52, 53). As the population grows, the aging process accelerates and the westernized lifestyles become more prevalent, in the coming years the incidence of pancreatic cancer is likely to continue to rise. Currently, surgery treatment is the only promising method for pancreatic cancer, usually followed by postoperative adjuvant radiotherapy and chemotherapy, and the survival rate of five year in patients is only 30% (54). Exocrine cell tumors account for 95% of pancreatic cancer cases, and the most common of these is PDAC. However, PDAC is characterized by early invasive metastases, with more than 50% of patients presenting with distant metastases, only 15%-20% of patients with PDAC can perform the surgery and most surgical patients also experience metastases within four years (55, 56). At present, the survival status of pancreatic cancer patients is not optimistic, and the treatment choices are limited. The median overall survival (mOS) of patients with advanced pancreatic cancer is only 11.1 months (41), so there is an imperative need to find new targets and new therapies to bring new hope to patients. In both pancreatic adenocarcinoma and its metastases, claudin18.2 is heavily expressed. Patients who have high expression of claudin18 live longer than individuals without this target (57). So, at present, the research on claudin18 monoclonal antibodies, bispecific antibodies, antibody-drug conjugates, and CAR-T cell therapy are in full swing, which may offer fresh ideas to the treatment of pancreatic cancer.

### 4.1 Monoclonal antibodies – the potential of Claudin18.2 is emerging

#### 4.1.1 Zolbetuximab (IMAB362)

Zolbetuximab (IMAB362) is one of monoclonal antibody drugs targeting Claudin 18.2. It can trigger ADCC and CDC by specifically connecting to Claudin18.2 on the surface of tumor cells, which may result in tumor cell lysis and death. The extent of cytotoxic effects induced by Zolbetuximab was found to be related to the expression of Claudin18.2 on the surface of cells (58). The study (NCT03816163) to evaluate the safety and effectiveness of the combination of Nab-Paclitaxel and Gemcitabine (Nab-P + GEM) with Zolbetuximab (IMAB362) as the first-line therapy for patients with metastatic pancreatic adenocarcinoma who are also Claudin18.2 positive is ongoing (50). The study is predicted to include 369 pancreatic cancer patients with high expression of Claudin18.2 (IHC staining intensity ≥75%). In order to assess the safety and tolerability of Zolbetuximab + GN, the trial involved a safety lead-in that enrolled 3-12 patients. And after 28 days, dose-limiting toxicities (DLTs) will be evaluated. About 357 patients will be assigned 2:1 randomly to receive either GN alone on Days 1, 8, and 15 of each cycle or Zolbetuximab Q2W on Days 1 and 15 plus GN on Days 1, 8, and 15 of each cycle, according to the recommended phase 2 dosage (RP2D), which was confirmed during the safety lead-in period. Liver metastases and ECOG performance status will be used to determine the randomization process. Patients will have the baseline MRI or CT every eight weeks until the beginning of another systemic anticancer treatment, or until the period that investigators assessed the disease progression, whichever occurs first. The main goals are to ascertain the safety and tolerability of Zolbetuximab + GN, and whether the treatment with Zolbetuximab + GN compared to GN alone improves overall survival (OS). Progression-free survival (PFS), objective response rate (ORR), disease control rate (DCR), pharmacokinetics, duration of response (DOR), and health-related quality of life are secondary endpoints. We are now looking forward to the final results of this experiment. Up to now, Zolbetuximab (IMAB362) has been approved for the first-line treatment for metastatic pancreatic cancer in China, and is expected to become the first marketed Claudin18.2 monoclonal antibody in the world.

#### 4.1.2 TST001

TST001 is a monoclonal antibody targeting Claudin18.2 that was developed globally following IMAB362. Compared to IMAB362, TST001 exhibits better affinity and Fc fragment receptor (FcR) binding activity because of its enhanced natural killer cell-mediated ADCC and fewer fucose content (59). Preclinical studies found that TST001 shows strong anti-tumor abilities in tumor models, with moderate to high levels of Claudin18.2 expression and synergistic anti-cancer effects with immune checkpoint inhibitors (ICIs) (60). The study has found that the combination of TST001 (Osemitamab) and atezolizumab has better anti-tumor activity than the single agent (61). There is an ongoing trial (NCT04396821) about TST001 to study the safety and tolerability in advanced or metastatic solid tumors. In cohort C of the trial, subjects with previously untreated, locally advanced, unresectable, or metastatic pancreatic adenocarcinoma will be enrolled. These participants will be given TST001 at a dose of 2 mg/kg or 4 mg/kg Q2W together with albumin-bound paclitaxel AND gemcitabine. The key observational indicators comprise the frequency and severity of adverse events, maximum tolerated dosage, safety and tolerability. And the secondary observational indicators include immunogenicity, PFS, ORR, DOR et al. We are awaiting the latest research progress.

#### 4.1.3 AB011

A humanized recombinant monoclonal antibody injection against Claudin18.2 is known as AB011. which is the first monoclonal antibody against Claudin18.2 independently developed in China and the first humanized monoclonal antibody against this target in the world. Currently, AB011 is in the phase I clinical trial (NCT04400383) for the treatment of Claudin18.2 positive solid tumors (pancreatic adenocarcinoma, gastric cancer, and solid tumors). The study is consisted of two phases: the first phase is a single treatment and the second phase is a combination therapy. From Aug 2020 to Aug 2021, in Part 1, 14 patients in the dose-escalation stage were given AB011 at different dosage and 21 patients in the dose-expansion stage (11 at 30 mg/kg, 10 at 20 mg/kg). 77.1% have had at least two lines of treatment before. Twelve subjects (12/46, 46.2%) of GC/gastroesophageal junction (GEJ) adenocarcinoma had three or more metastatic organs, of which fifteen (56.7%) had peritoneal metastases. Six out of nine (66.7%) pancreatic cancer subjects had two or more metastatic organs. Eight patients (3 in 20 mg/kg, 5 in 30 mg/kg) suffered treatment-related adverse events (TRAEs) of grade 3, with grade 1-2 accounting for the majority of TRAEs. One case of DLT (grade 3 dyspnea) was reported in the 30 mg/kg group. Twelve patients achieved disease control out of the twenty patients with evaluable disease and at least one tumor assessment, and one GC without target lesions was shown to be complete response (CR) (51). There are some other monoclonal antibodies against Claudin18.2 that are being studied, like ASKB589 (NCT04632108), M108 (NCT04894825), MIL93 (NCT04671875), NBL-015 (NCT05153096), ZL-1211 (NCT05065710).

### 4.2 Bispecific antibodies – Claudin 18.2 is a strong combination

Two distinct epitopes on the same or different antigens are bound by bispecific antibodies. Bispecific antibodies exert activity that exceeds that of beyond natural antibodies due to the dual specificity of soluble or cell surface antigens, which may present additional therapeutic application potential (62). Currently, bispecific antibodies that have entered clinical trials include: Claudin18.2/CD3 (NCT04260191) (44), Claudin18.2/4-1BB (NCT05839106) (45), Claudin18.2/PD-L1 (NCT04856150) et al. These bispecific antibodies have stronger specificity and lower off-target toxicity than some monoclonal antibodies, and have more therapeutic potential for gastric and pancreatic cancers. AZD5863 is a bispecific antibody which targets Claudin18.2 and CD3 T cells. The safety, pharmacokinetics, pharmacodynamics, and effectiveness of AZD5863 in adult patients who have advanced or metastatic solid tumors are being investigated in the trial (NCT06005493). In this experiment, the AZD5863 will be infused in two different forms, intravenous and subcutaneous, to observe the number of adverse reactions and ORR. Clinical studies of other bispecific antibodies are ongoing, for example, IBI389 (NCT05164458), QLS31905 (NCT05278832), SG1906 (NCT05857332) et al.

### 4.3 Antibody-drug conjugates – Claudin 18.2 is getting better

#### 4.3.1 CMG901

The most common form of antibody-drug conjugates (ADC) is monoclonal antibodies (mAbs) linked to cytotoxic medicines via chemical linkers by covalent bonds. Because of the benefits of strong killing power and high specificity targeting ability, it removes cancer cells effectively, making it a hotspot in the production of anticancer medications (63). CMG901 is the first ADC drug for Claudin18.2 in the world, which has shown strong anti-cancer efficacy through CDC, ADCC, and MMAE-mediated cytotoxicity with bystander killing. The phase I trial (NCT04805307) in patients who have advanced solid tumors is ongoing. As of August 4, 2022, CMG901 had been administered to 27 participants (14 patients with pancreatic cancer and 13 patients with gastric/GEJ cancer) at doses of 3.4 mg/kg. The median line of prior systemic treatment was 2, with the range of 1 to 5. 3/27 (11.1%) of the patients experienced grade 3 adverse events (AEs) as a result of the medicine. There were no documented grade 4 or 5 AEs linked to the drug. MTD wasn’t accomplished. The ORR and DCR in patients with gastric/GEJ cancer who were positive for Claudin18.2 were 75.0% and 100%, respectively. In the 2.6, 3.0, and 3.4 mg/kg Q3W dosage groups, ORR was 100%. The mOS and mPFS had not yet been reached. Patients exhibited little exposure to unconjugated MMAE systemically, according to the data. The results will be reported on an ongoing basis (43).

#### 4.3.2 LM-302

LM-302 can specifically target Claudin18.2-positive tumor cells and enter tumor cells through endocytosis, releasing small molecule toxins, thus exerting anti-tumor effects. Preclinical studies of LM-302 (TPX-4589) have shown favorable safety profile and activity *in vitro* and *in vivo*, including good tumor suppression and decreasing tumor size in tumor models with high- or low-expression of Claudin18.2 (64). The Food and Drug Administration (FDA) has declared the medicine as the orphan drug (ODD) in 2021 for pancreatic cancer, gastric cancer and gastric junction cancer. Currently, experiments on LM-302 are underway, like NCT05161390, NCT05994001, NCT05934331, NCT05001516.

#### 4.3.3 EO-3021/SYSA1801

A monoclonal antibody specific to the Claudin18.2 target and an MMAE payload site-specifically attached by cleavable linkers make up EO-3021/SYSA1801. The trial found that EO-3021 exhibits antitumor activity by inducing tumor regression with the single dosage in the models of gastric cancers, pancreatic cancers, and lung cancers with low, medium, and high Claudin18.2 expression (65). SYSA1801 is being assessed in patients with advanced solid tumors who are Claudin18.2 positive for the safety, anticancer efficacy, tolerability, and pharmacokinetics in the phase 1 trial (NCT05009966).

### 4.4 CAR-T cell – there is a breakthrough targeting Claudin18.2

#### 4.4.1 CT041

Artificial receptor molecule made by genetic engineering technology, CAR can direct lymphocytes, most often T cells, to identify and destroy cells that express particular target antigens. Regardless of the major histocompatibility complex (MHC) receptor, the CAR connects to the target antigens that presented on the cell surface, leading to strong T cell activity and powerful anti-tumor effect (66). As the first CAR-T cell drug targeting Claudin18.2 in the world, CT041 appeared in 2019, and its remarkable efficacy shows good prospects for the treatment of digestive system tumors. Twelve patients-seven with gastric cancer and five with pancreatic cancer-were offered with CAR-positive T cell infusions in the open-label, single-arm, phase I study (NCT03159819). The results were found that among the 11 evaluable individuals one had CR who had a gastric adenocarcinoma, three had partial response (PR) (two gastric and one pancreatic adenocarcinoma), five had stable disease (SD), and two had progressive disease (PD). There was a 33.3% ORR, with a 130 days mPFS. This suggests that patients with advanced gastric cancer and pancreatic cancer may benefit from CAR- Claudin18.2 T cells (46). Qi C et al. reported two cases (NCT04581473 and NCT03874897) of CT041 in patients who had failed standard therapy with Claudin18.2 positive metastatic pancreatic cancer (48). After lymphocyte depletion, CAR-T cells were injected in the example 1, where the expression of Claudin18.2 was 2+, 70%. On day 1, there was grade 1 cytokine release syndrome (CRS) appeared, which was handled successfully by tocilizumab. PR was reached with a significant reduction in lung metastases, based on RECIST v1.1. There was a rise in CD8^+^ T cells and Treg cells and a decrease in CD4^+^ T cells and B cells. The IHC in the example 2 for Claudin18.2 was 3+, 60%. After that, Claudin18.2 CAR-T cells were given. The patient had grade 2 CRS, which was treated by tocilizumab. In addition, CR was attained in the lung metastatic target lesions. From peripheral blood, similar results that there were rises in both CD8^+^ T cells and Treg cells. Furthermore, decreasing transforming growth factor-β1 (TGF-β1) and increasing interleukin-8 (IL-8) were also reported. Until the final follow-up on July 18, 2023, the tumor was remained under control. In the interim results of a phase I trial (NCT03874897), the total of 37 patients with metastatic gastrointestinal tumors (five patients with pancreatic cancer) were included to receive CT041 infusion. For all patients, the ORR was 48.6% (95% confidence interval (CI); 31.9, 65.6) and the DCR was 73.0% (95% CI; 55.9, 86.2). The mPFS was 3.7 months (95% CI; 2.6, 5.4), and the OS rate was 80.1% (95% CI; 62.5, 90.0) at 6 months. In pancreatic cancer and other cancers group (n=9), 2 had PR, 4 had SD. The ORR and DCR were 22.2% (95% CI; 2.8,60.0) and 66.7% (95% CI; 29.9,92.5), respectively. The mPFS was 2.6 months (95% CI; 1.8,3.5) (47). In the single-arm, open-label, phase Ib study (NCT04404595), at the end of February 15, 2022, eleven participators (5 had gastric cancer and 6 had pancreatic cancer) were treated with CT041 at the dose between 2.5 and 4 × 10^8^ cells. Two patients with pancreatic cancer were in stable disease with reduced tumor size, and there were also 3 patients with pancreatic cancer who progressed on the disease. The ORR was 37.5% (3/8) in patients (49). There are also experiments (NCT05911217) on CT041 that are in progress. The studies above have demonstrated that CT041, a Claudin18.2-targeted CAR-T cell drug, has good anti-tumor activity in advanced gastrointestinal tumors, including pancreatic cancer, and is safe and reliable.

#### 4.4.2 Other CAR-T drugs

LY011 is one of the third generation of CAR-T cell products against Claudin18.2. Currently, two phase I trials (NCT04977193, NCT04966143) are being conducted in patients with Claudin18.2 positive pancreatic cancer and advanced gastric cancer, and as of now the results have not been officially reported. In addition, LB1908 (NCT05539430), HEC-016 (NCT05277987), KD-496 (NCT05583201), and CT048 (NCT05393986) have entered clinical trials for the treatment of pancreatic cancer, gastric cancer, and other solid tumors to verify their safety, tolerability and efficacy. The results of these clinical studies are awaiting.

More and more evidence suggest that pancreatic cancer has the firm barrier, deep invasion of immunosuppressive cells, and deficiency of effector T cells in the suppressive tumor microenvironment, which is considered as the major factor in chemotherapy resistance, immunotherapy insensitivity, and recurrence and metastasis (67). Therefore, in order to improve the anti-tumor effect of Claudin18.2-targeted CAR-T cell in the pancreatic cancer, a feasible strategy may be to remodel the immune microenvironment. This also means that there is still room for further advancement and promotion of the clinical use of Claudin18.2 targets. In addition, the combination of other immunomodulators, targeted tumor angiogenesis drugs, or improvement of CAR-T cells may also provide more effective treatments for pancreatic cancer patients.

## 5 Summary and outlook

In summary, Claudin18.2 is expected to become a “dark horse” target due to its high selectivity for pancreatic cancer in the future, and its targeted drugs include monoclonal antibodies, bispecific antibodies, ADC, CAR-T and other mainstream directions. And a number of ongoing clinical trials are also expected to offer more choices for patients who have Claudin18.2 positive pancreatic cancer. At present, the research and development of medicine which target Claudin18.2 is mostly based on IHC, but there are differences in the detection antibodies used by different institutions and the patient populations included, resulting in differences in the positive rate of Claudin18.2. Therefore, accurate diagnosis and multi-dimensional screening methods are essential for the detection of Claudin18.2-positive patients, while screening for beneficiary populations is also a key issue in accurately defining Claudin18.2-positive patients. But overall, targeted Claudin18.2 therapy has a “bright future”.

## Author contributions

QX: Writing – original draft, Writing – review & editing. CJ: Writing – original draft, Writing – review & editing. YO: Writing – original draft, Writing – review & editing. CZ: Visualization, Writing – review & editing. YJ: Validation, Writing – review & editing.
